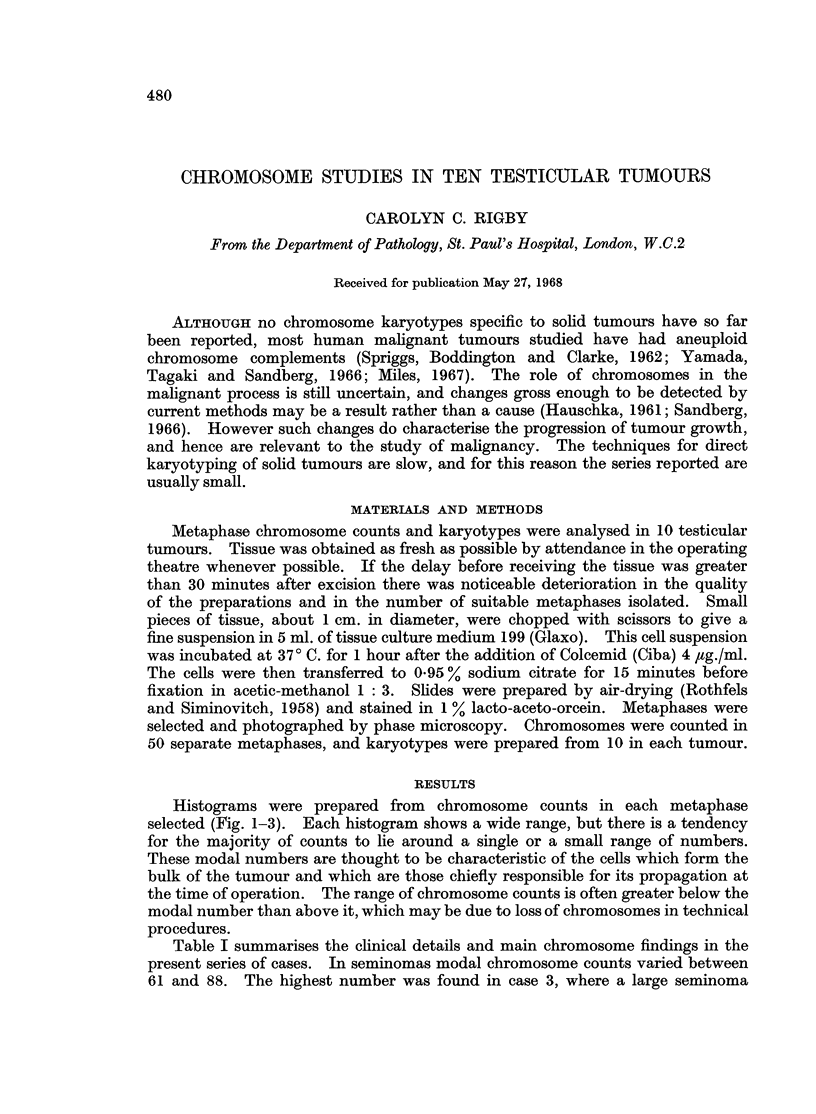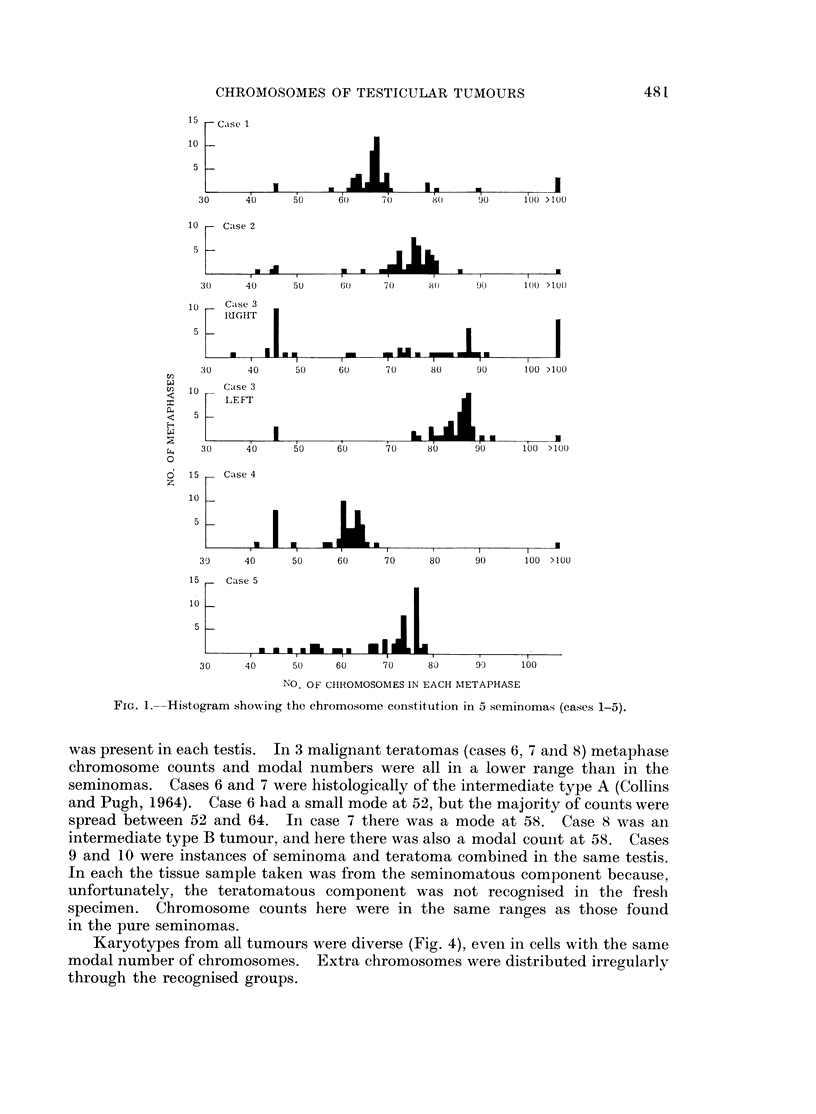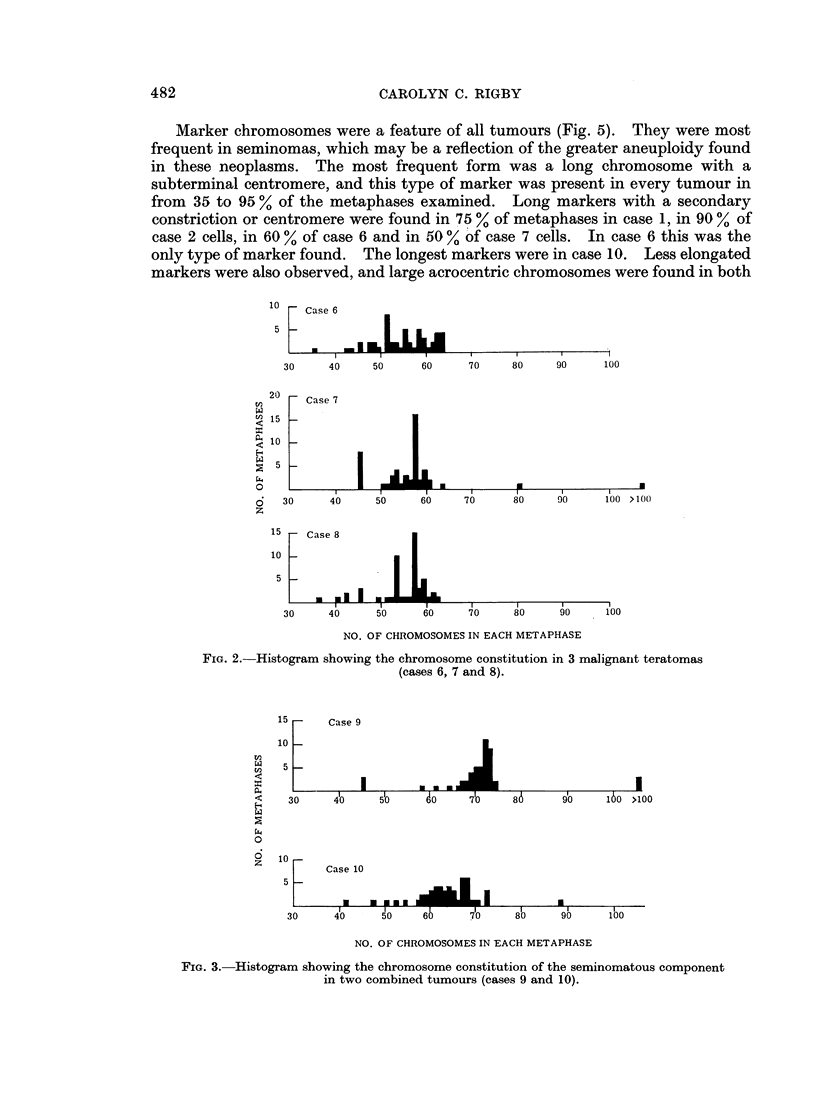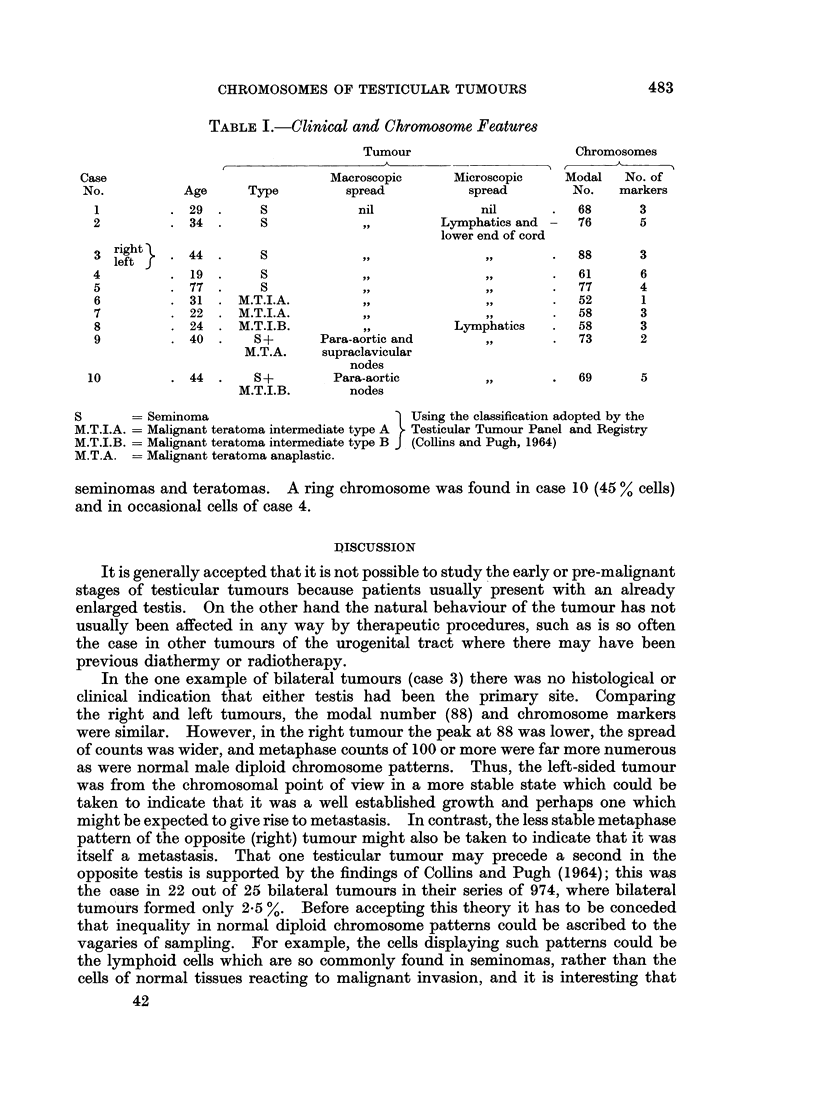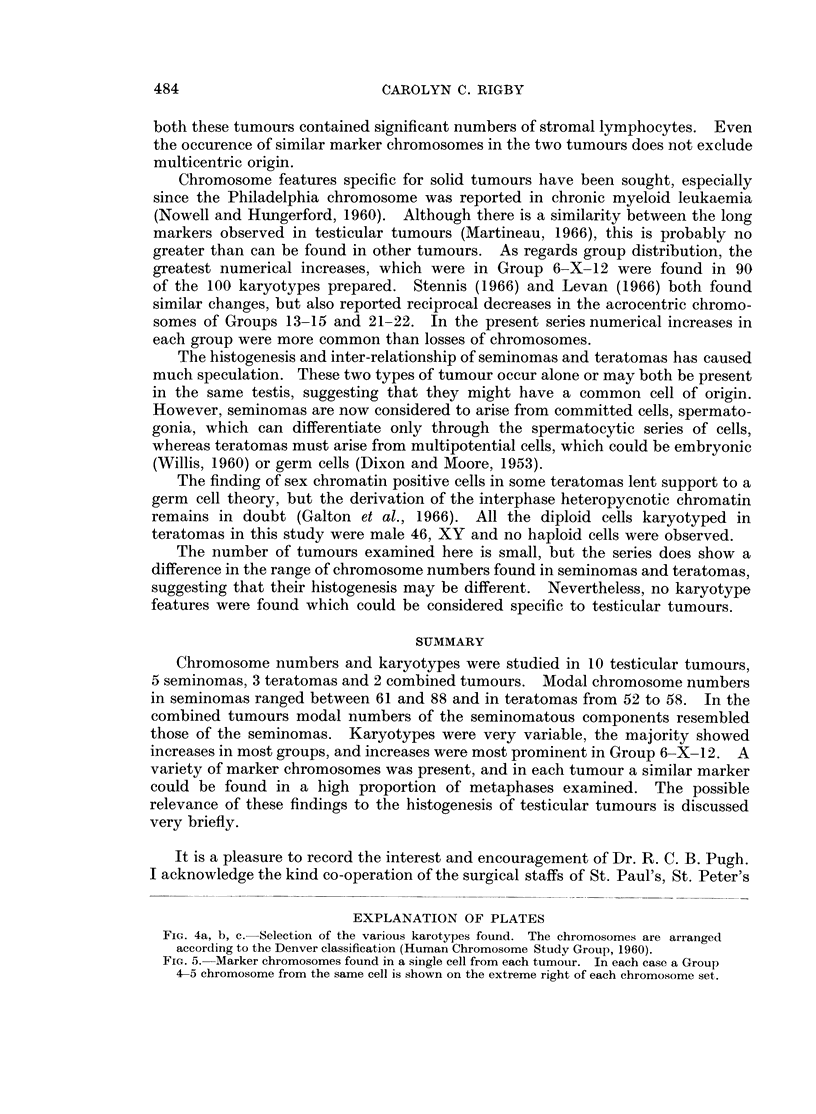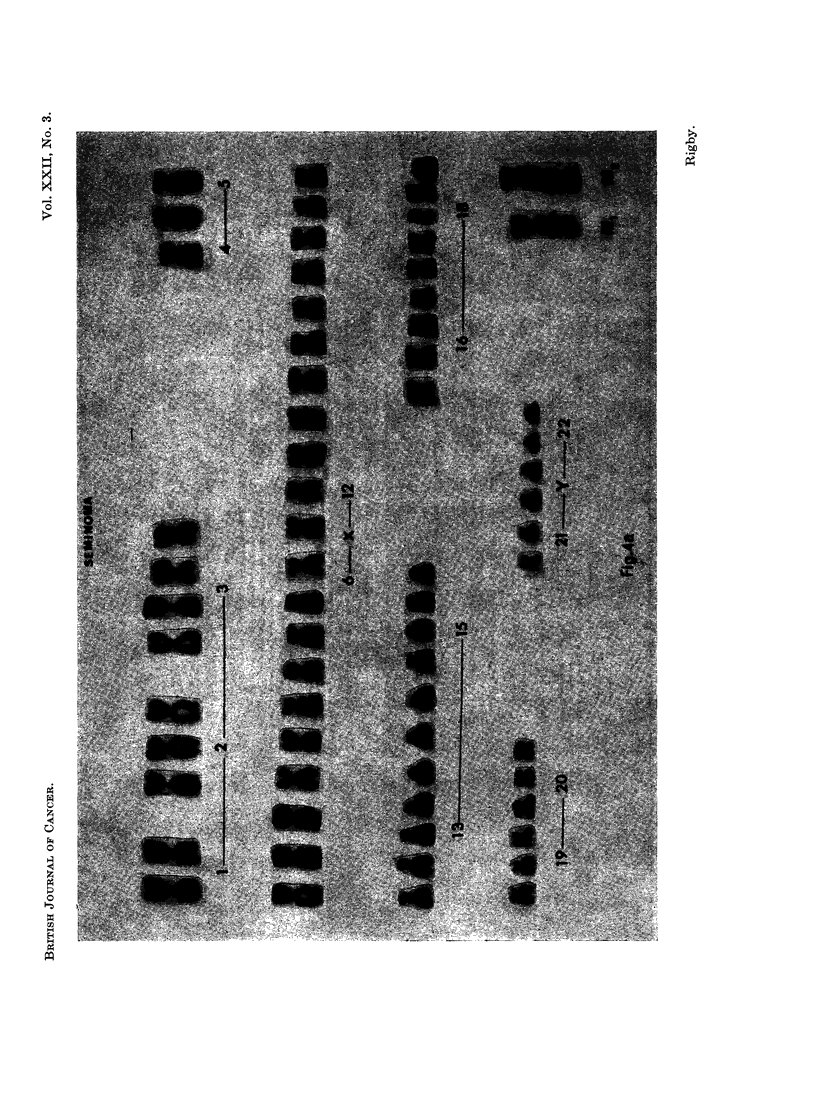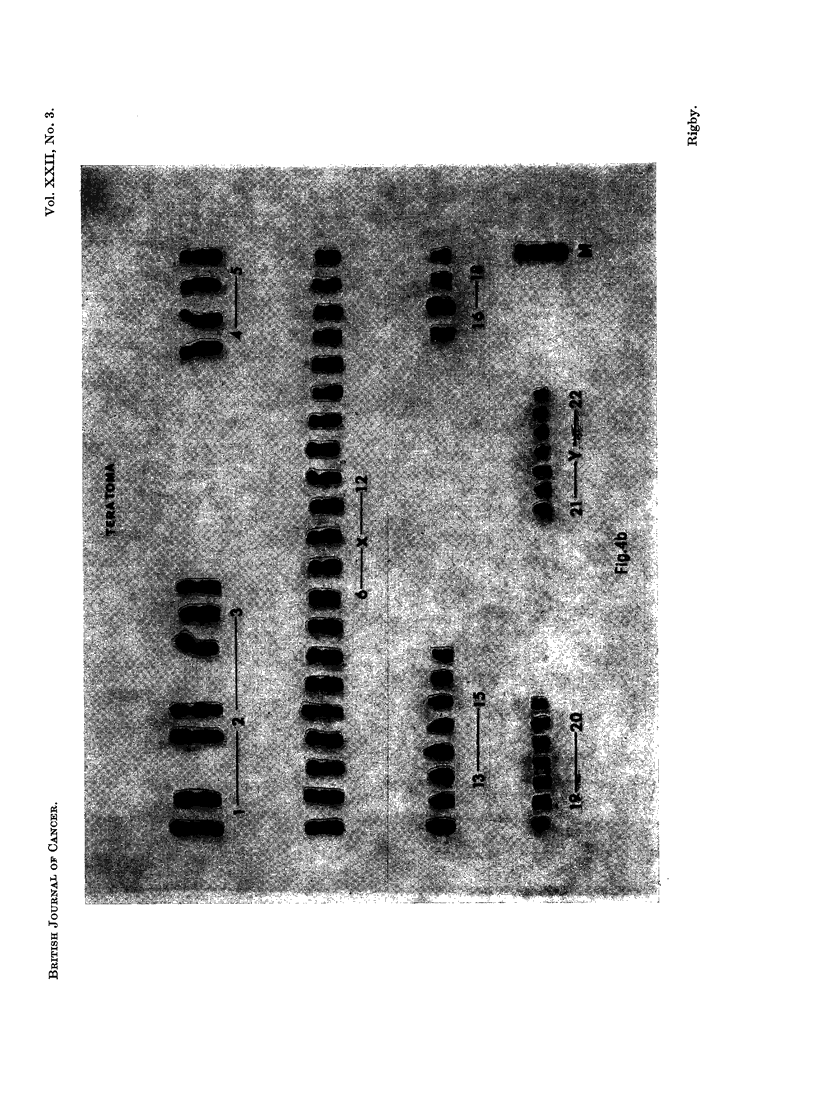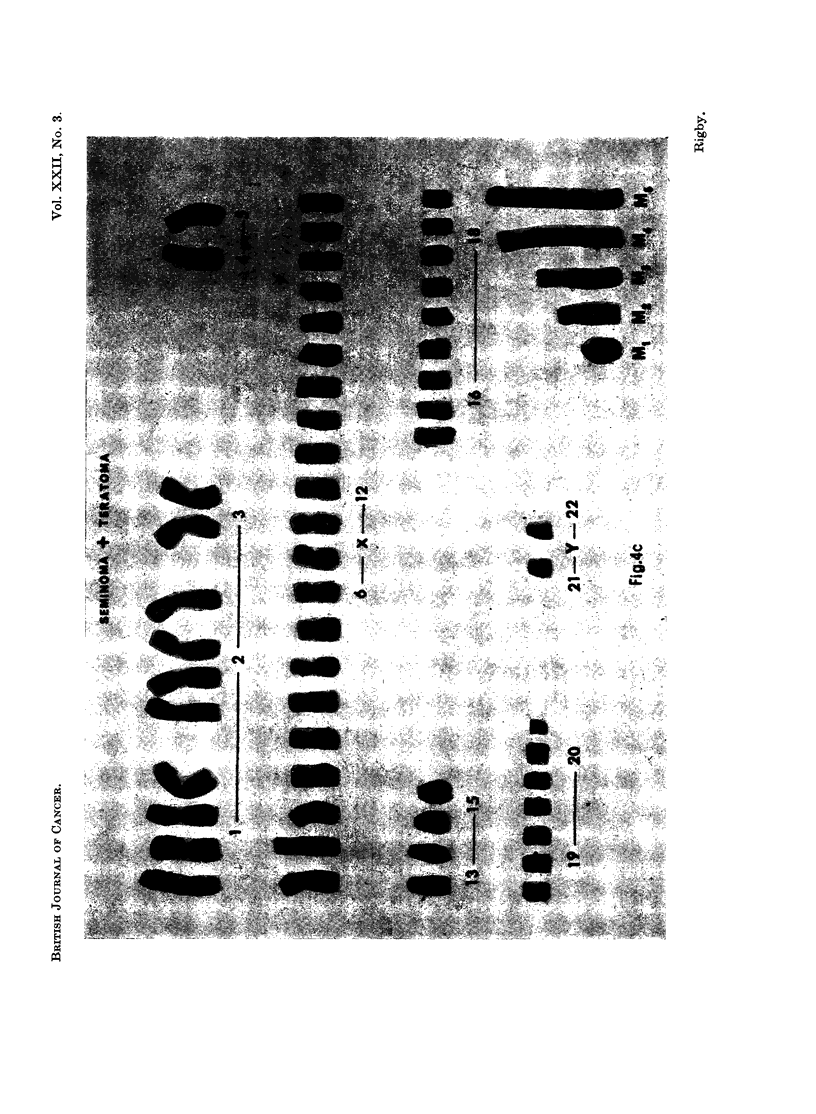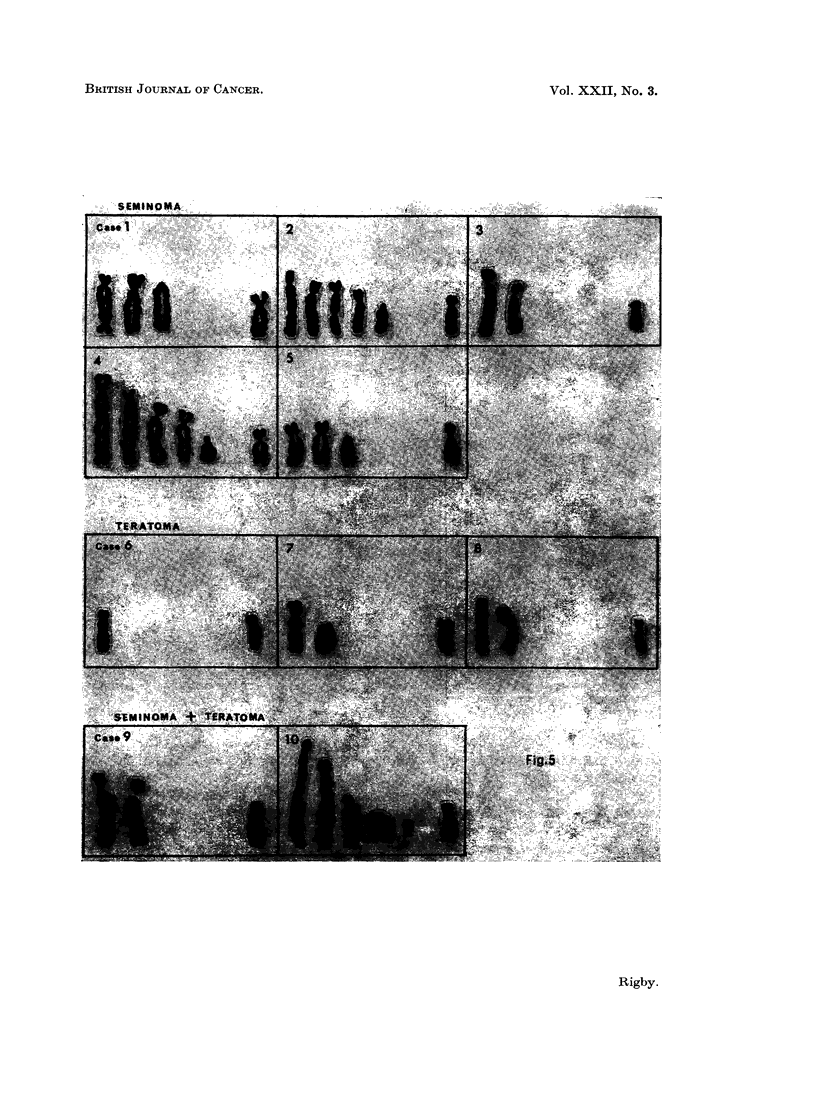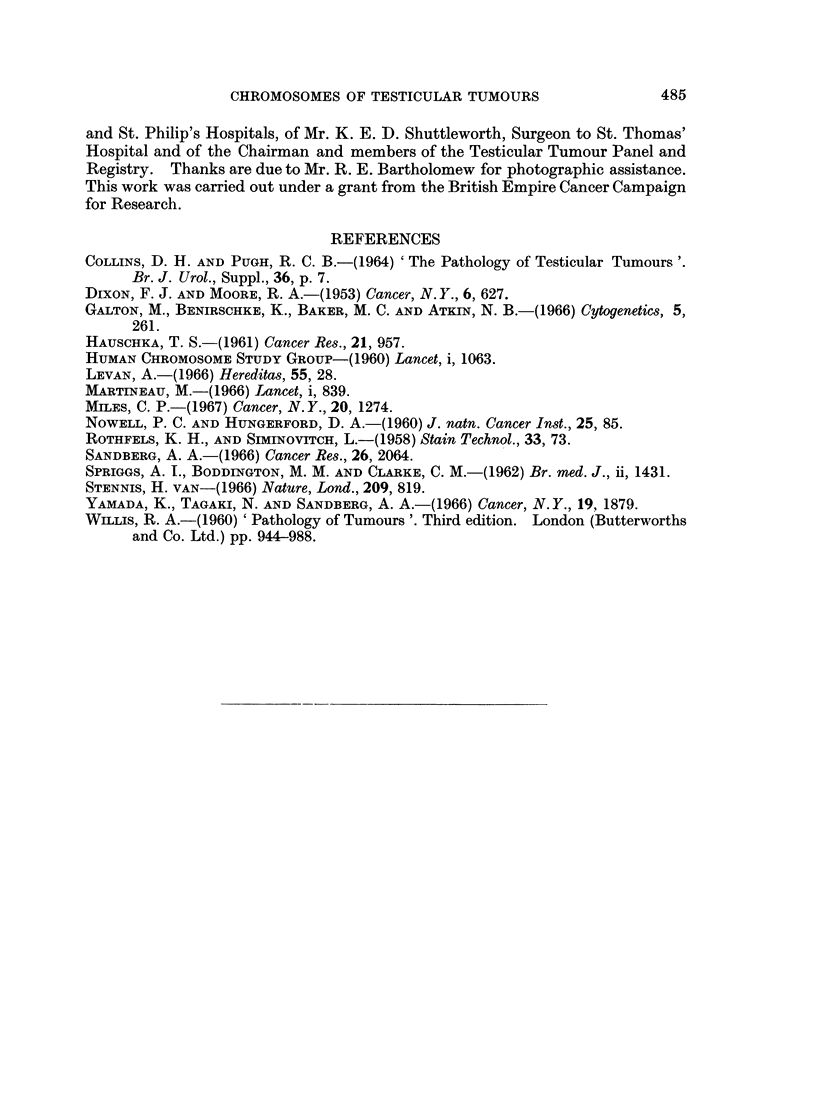# Chromosome studies in ten testicular tumours.

**DOI:** 10.1038/bjc.1968.56

**Published:** 1968-09

**Authors:** C. C. Rigby

## Abstract

**Images:**


### CHROMOSOME STUDIES IN TEN TESTICULAR TUMOURS

CAROLYN C. RIGBY

*From the Department of Pathology, St. Paul's Hospital, London, W.C.2*

Received for publication May 27, 1968

ALTHOUGH no chromosome karyotypes specific to solid tumours have so far been reported, most human malignant tumours studied have had aneuploid chromosome complements (Spriggs, Boddington and Clarke, 1962; Yamada, Tagaki and Sandberg, 1966; Miles, 1967). The role of chromosomes in the malignant process is still uncertain, and changes gross enough to be detected by current methods may be a result rather than a cause (Hauschka, 1961; Sandberg, 1966). However such changes do characterise the progression of tumour growth, and hence are relevant to the study of malignancy. The techniques for direct karyotyping of solid tumours are slow, and for this reason the series reported are usually small.

### MATERIALS AND METHODS

Metaphase chromosome counts and karyotypes were analysed in 10 testicular tumours. Tissue was obtained as fresh as possible by attendance in the operating theatre whenever possible. If the delay before receiving the tissue was greater than 30 minutes after excision there was noticeable deterioration in the quality of the preparations and in the number of suitable metaphases isolated. Small pieces of tissue, about 1 cm. in diameter, were chopped with scissors to give a fine suspension in 5 ml. of tissue culture medium 199 (Glaxo). This cell suspension was incubated at 37° C. for 1 hour after the addition of Colcemid (Ciba) 4 $\mu$g./ml. The cells were then transferred to 0·95% sodium citrate for 15 minutes before fixation in acetic-methanol 1 : 3. Slides were prepared by air-drying (Rothfels and Siminovitch, 1958) and stained in 1% lacto-aceto-orcein. Metaphases were selected and photographed by phase microscopy. Chromosomes were counted in 50 separate metaphases, and karyotypes were prepared from 10 in each tumour.

### RESULTS

Histograms were prepared from chromosome counts in each metaphase selected (Fig. 1–3). Each histogram shows a wide range, but there is a tendency for the majority of counts to lie around a single or a small range of numbers. These modal numbers are thought to be characteristic of the cells which form the bulk of the tumour and which are those chiefly responsible for its propagation at the time of operation. The range of chromosome counts is often greater below the modal number than above it, which may be due to loss of chromosomes in technical procedures.

Table I summarises the clinical details and main chromosome findings in the present series of cases. In seminomas modal chromosome counts varied between 61 and 88. The highest number was found in case 3, where a large seminoma

FIG. 1.—Histogram showing the chromosome constitution in 5 seminomas (cases 1–5).

was present in each testis. In 3 malignant teratomas (cases 6, 7 and 8) metaphase chromosome counts and modal numbers were all in a lower range than in the seminomas. Cases 6 and 7 were histologically of the intermediate type A (Collins and Pugh, 1964). Case 6 had a small mode at 52, but the majority of counts were spread between 52 and 64. In case 7 there was a mode at 58. Case 8 was an intermediate type B tumour, and here there was also a modal count at 58. Cases 9 and 10 were instances of seminoma and teratoma combined in the same testis. In each the tissue sample taken was from the seminomatous component because, unfortunately, the teratomatous component was not recognised in the fresh specimen. Chromosome counts here were in the same ranges as those found in the pure seminomas.

Karyotypes from all tumours were diverse (Fig. 4), even in cells with the same modal number of chromosomes. Extra chromosomes were distributed irregularly through the recognised groups.

Marker chromosomes were a feature of all tumours (Fig. 5). They were most frequent in seminomas, which may be a reflection of the greater aneuploidy found in these neoplasms. The most frequent form was a long chromosome with a subterminal centromere, and this type of marker was present in every tumour in from 35 to 95% of the metaphases examined. Long markers with a secondary constriction or centromere were found in 75% of metaphases in case 1, in 90% of case 2 cells, in 60% of case 6 and in 50% of case 7 cells. In case 6 this was the only type of marker found. The longest markers were in case 10. Less elongated markers were also observed, and large acrocentric chromosomes were found in both

Fig. 2.—Histogram showing the chromosome constitution in 3 malignant teratomas (cases 6, 7 and 8).

Fig. 3.—Histogram showing the chromosome constitution of the seminomatous component in two combined tumours (cases 9 and 10).

TABLE I.—*Clinical and Chromosome Features*

| Case No. | | Age | Tumour: Type | Tumour: Macroscopic spread | Tumour: Microscopic spread | Chromosomes: Modal No. | Chromosomes: No. of markers |
|---|---|---|---|---|---|---|---|
| 1 | | 29 | S | nil | nil | 68 | 3 |
| 2 | | 34 | S | „ | Lymphatics and lower end of cord | 76 | 5 |
| 3 | right } left } | 44 | S | „ | „ | 88 | 3 |
| 4 | | 19 | S | „ | „ | 61 | 6 |
| 5 | | 77 | S | „ | „ | 77 | 4 |
| 6 | | 31 | M.T.I.A. | „ | „ | 52 | 1 |
| 7 | | 22 | M.T.I.A. | „ | „ | 58 | 3 |
| 8 | | 24 | M.T.I.B. | „ | Lymphatics | 58 | 3 |
| 9 | | 40 | S+ M.T.A. | Para-aortic and supraclavicular nodes | „ | 73 | 2 |
| 10 | | 44 | S+ M.T.I.B. | Para-aortic nodes | „ | 69 | 5 |

S = Seminoma
M.T.I.A. = Malignant teratoma intermediate type A
M.T.I.B. = Malignant teratoma intermediate type B
} Using the classification adopted by the Testicular Tumour Panel and Registry (Collins and Pugh, 1964)
M.T.A. = Malignant teratoma anaplastic.

seminomas and teratomas. A ring chromosome was found in case 10 (45 % cells) and in occasional cells of case 4.

### DISCUSSION

It is generally accepted that it is not possible to study the early or pre-malignant stages of testicular tumours because patients usually present with an already enlarged testis. On the other hand the natural behaviour of the tumour has not usually been affected in any way by therapeutic procedures, such as is so often the case in other tumours of the urogenital tract where there may have been previous diathermy or radiotherapy.

In the one example of bilateral tumours (case 3) there was no histological or clinical indication that either testis had been the primary site. Comparing the right and left tumours, the modal number (88) and chromosome markers were similar. However, in the right tumour the peak at 88 was lower, the spread of counts was wider, and metaphase counts of 100 or more were far more numerous as were normal male diploid chromosome patterns. Thus, the left-sided tumour was from the chromosomal point of view in a more stable state which could be taken to indicate that it was a well established growth and perhaps one which might be expected to give rise to metastasis. In contrast, the less stable metaphase pattern of the opposite (right) tumour might also be taken to indicate that it was itself a metastasis. That one testicular tumour may precede a second in the opposite testis is supported by the findings of Collins and Pugh (1964); this was the case in 22 out of 25 bilateral tumours in their series of 974, where bilateral tumours formed only 2·5 %. Before accepting this theory it has to be conceded that inequality in normal diploid chromosome patterns could be ascribed to the vagaries of sampling. For example, the cells displaying such patterns could be the lymphoid cells which are so commonly found in seminomas, rather than the cells of normal tissues reacting to malignant invasion, and it is interesting that both these tumours contained significant numbers of stromal lymphocytes. Even the occurence of similar marker chromosomes in the two tumours does not exclude multicentric origin.

Chromosome features specific for solid tumours have been sought, especially since the Philadelphia chromosome was reported in chronic myeloid leukaemia (Nowell and Hungerford, 1960). Although there is a similarity between the long markers observed in testicular tumours (Martineau, 1966), this is probably no greater than can be found in other tumours. As regards group distribution, the greatest numerical increases, which were in Group 6–X–12 were found in 90 of the 100 karyotypes prepared. Stennis (1966) and Levan (1966) both found similar changes, but also reported reciprocal decreases in the acrocentric chromosomes of Groups 13–15 and 21–22. In the present series numerical increases in each group were more common than losses of chromosomes.

The histogenesis and inter-relationship of seminomas and teratomas has caused much speculation. These two types of tumour occur alone or may both be present in the same testis, suggesting that they might have a common cell of origin. However, seminomas are now considered to arise from committed cells, spermatogonia, which can differentiate only through the spermatocytic series of cells, whereas teratomas must arise from multipotential cells, which could be embryonic (Willis, 1960) or germ cells (Dixon and Moore, 1953).

The finding of sex chromatin positive cells in some teratomas lent support to a germ cell theory, but the derivation of the interphase heteropycnotic chromatin remains in doubt (Galton *et al.*, 1966). All the diploid cells karyotyped in teratomas in this study were male 46, XY and no haploid cells were observed.

The number of tumours examined here is small, but the series does show a difference in the range of chromosome numbers found in seminomas and teratomas, suggesting that their histogenesis may be different. Nevertheless, no karyotype features were found which could be considered specific to testicular tumours.

### SUMMARY

Chromosome numbers and karyotypes were studied in 10 testicular tumours, 5 seminomas, 3 teratomas and 2 combined tumours. Modal chromosome numbers in seminomas ranged between 61 and 88 and in teratomas from 52 to 58. In the combined tumours modal numbers of the seminomatous components resembled those of the seminomas. Karyotypes were very variable, the majority showed increases in most groups, and increases were most prominent in Group 6–X–12. A variety of marker chromosomes was present, and in each tumour a similar marker could be found in a high proportion of metaphases examined. The possible relevance of these findings to the histogenesis of testicular tumours is discussed very briefly.

It is a pleasure to record the interest and encouragement of Dr. R. C. B. Pugh. I acknowledge the kind co-operation of the surgical staffs of St. Paul's, St. Peter's

---

### EXPLANATION OF PLATES

FIG. 4a, b, c.—Selection of the various karotypes found. The chromosomes are arranged according to the Denver classification (Human Chromosome Study Group, 1960).

FIG. 5.—Marker chromosomes found in a single cell from each tumour. In each case a Group 4–5 chromosome from the same cell is shown on the extreme right of each chromosome set.

Fig.4a

Rigby.

Fig.4b

Rigby.

SEMINOMA + TERATOMA
1 —— 2 —— 3
4 —— 5
6 — X — 12
13 —— 15
16 —— 18
19 —— 20
21 — Y — 22
Fig.4c
$M_1$ $M_2$ $M_3$ $M_4$ $M_5$

SEMINOMA

Case 1

2

3

4

5

TERATOMA

Case 6

7

8

SEMINOMA + TERATOMA

Case 9

10

Fig.5

and St. Philip's Hospitals, of Mr. K. E. D. Shuttleworth, Surgeon to St. Thomas' Hospital and of the Chairman and members of the Testicular Tumour Panel and Registry. Thanks are due to Mr. R. E. Bartholomew for photographic assistance. This work was carried out under a grant from the British Empire Cancer Campaign for Research.

### REFERENCES

Collins, D. H. and Pugh, R. C. B.—(1964) ' The Pathology of Testicular Tumours '. *Br. J. Urol.*, Suppl., **36**, p. 7.
Dixon, F. J. and Moore, R. A.—(1953) *Cancer, N.Y.*, **6**, 627.
Galton, M., Benirschke, K., Baker, M. C. and Atkin, N. B.—(1966) *Cytogenetics*, **5**, 261.
Hauschka, T. S.—(1961) *Cancer Res.*, **21**, 957.
Human Chromosome Study Group—(1960) *Lancet*, i, 1063.
Levan, A.—(1966) *Hereditas*, **55**, 28.
Martineau, M.—(1966) *Lancet*, i, 839.
Miles, C. P.—(1967) *Cancer, N.Y.*, **20**, 1274.
Nowell, P. C. and Hungerford, D. A.—(1960) *J. natn. Cancer Inst.*, **25**, 85.
Rothfels, K. H., and Siminovitch, L.—(1958) *Stain Technol.*, **33**, 73.
Sandberg, A. A.—(1966) *Cancer Res.*, **26**, 2064.
Spriggs, A. I., Boddington, M. M. and Clarke, C. M.—(1962) *Br. med. J.*, ii, 1431.
Stennis, H. van—(1966) *Nature, Lond.*, **209**, 819.
Yamada, K., Tagaki, N. and Sandberg, A. A.—(1966) *Cancer, N.Y.*, **19**, 1879.
Willis, R. A.—(1960) ' Pathology of Tumours '. Third edition. London (Butterworths and Co. Ltd.) pp. 944–988.